# Practical Approach and Safety of Hyaluronic Acid Fillers

**DOI:** 10.1097/GOX.0000000000002172

**Published:** 2019-06-14

**Authors:** Rod J. Rohrich, Erica L. Bartlett, Erez Dayan

**Affiliations:** From the Dallas Plastic Surgery Institute, Dallas, Tex.

## Abstract

Soft-tissue filler use has grown considerably related to the increasing popularity of minimally invasive cosmetic procedures. Hyaluronic acid products are currently the most utilized soft-tissue fillers. Proper working knowledge of individual products, limitations to use, and anatomic principles can improve outcomes. Prevention is key to minimize complications; however, when present, complications must be managed methodically. Complications are categorized based on the timing of presentation and include early, late, and delayed. Vascular compromise and tissue necrosis are among the most devastating complications seen with filler use. Nodules can be related to an inflammatory or infectious etiology but should be distinguished as treatment varies. Hyaluronidase is mandatory to have available as a reversal agent for hyaluronic acid products and can be used in treatment for many complications and untoward sequela.

## Abstract 2

Supplemental Digital Content is available in the text.

## INTRODUCTION

Plastic surgery remains an innovative field with ongoing growth. Cosmetic procedures, both surgical and minimally invasive, continue to be in demand. Minimally invasive cosmetic procedures have increased 300% from 2000.^1^ Among the most utilized minimally invasive cosmetic procedures, soft-tissue fillers rank number 2, behind neuromodulators.^1^

There is a plethora of literature detailing the use and safety of soft-tissue fillers. However, new products are emerging and continue to diversity clinical application. The aim of this article is to describe the common hyaluronic acid (HA) soft-tissue fillers approved by the U.S. Food and Drug Administration (FDA) in the United States by their unique manufacturing processes, review injections by region, and discuss complications with a focus on patient safety.

## HISTORICAL PERSPECTIVE

Historically, the first soft-tissue fillers were derived from bovine collagen and were marketed as Zyderm (Allergan, Dublin, Ireland). Originally approved by the FDA in 1981, these products for soft-tissue augmentation were the original standard for which subsequent products were compared.^2^ Hypersensitivity reactions were common; thus, skin tests were required.^2,3^ Human collagens were marketed as CosmoDerm (Allergan) in 2003 to eliminate hypersensitivity but never gained as much popularity.^3,4^

During this time, there was a paradigm shift from injectable proteins to one of an injectable extracellular matrix form, HA.^5^ This was due, in part, to the enhanced understanding of facial aging, with goals not only to fill fine lines and wrinkles but also to volumize.^6^ Synthetic HA products have been used worldwide since the 1990s; however, the first formulation was introduced in the United States in 2003 as Restylane (Galderma S.A., Lausanne, Switzerland).

Unlike collagen, HA is consistent across species and has limited immunogenic potential. It also has a longer duration of effect, especially in more mobile areas of the face.^7^ HA also has a hydrating effect, which is beneficial in the aging face.^8^ The United States has been the slowest to gain widespread use, related to FDA control; however, globally, an abundance of HA-based soft-tissue fillers have been utilized. Adjuncts to HA fillers were similarly formulated during this time in hopes of having more substantial long-term effects, but these have not yet achieved as widespread use.

## RHEOLOGY

Rheology is defined as the study of flow-related properties.^9^ It reflects different manufacturing processes and physiochemical characteristics of the product.^10^ Clinically applied, understanding the specific properties of a filler can improve proper use. It guides appropriate placement and prevents complications. Rheologic properties are affected by the manufacturing technology of each product and the HA concentration, cross-linking, chain length, and particulate sizing (Table 1).^10–13^ The result is a unique gel with a particular texture. This gel will interact with tissue based on its viscoelastic characteristics. Knowledge of these viscoelastic properties guides physicians on appropriate product selection based on desired results.^14^

**Table 1.**
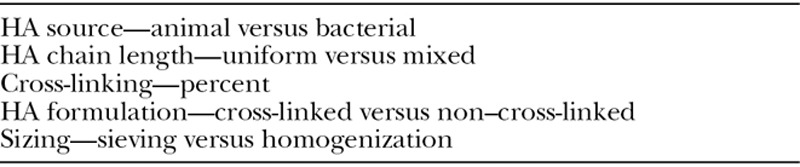
Factors Affecting Rheologic Properties

The most common descriptor for fillers is elastic modulus (*G*´). It is used to describe the firmness of a gel and represents the ability of a gel to resist deformation by an applied force.^9^ Generally speaking, gels with higher *G*´ have a better ability to resist dynamic forces and have more volumizing and support properties.^10^ They also require deeper placement. As HA concentration and cross-linking increase, so does the *G*´.^12^ As cross-linking increases so does water absorption. Water absorption is another factor that aids product selection. For example, regions such as lips respond favorably to products with high water absorption, whereas the tear trough does not. Cohesivity has also been identified as an important characteristic in filler selection. It is critical for gel integrity by maintaining its shape after implantation.^13^ Cohesivity contributes to tissue expansion, where *G*´ contributes to tissue projection. Both affect the “lifting” ability of a product but to different degrees (Table 2).

**Table 2.**
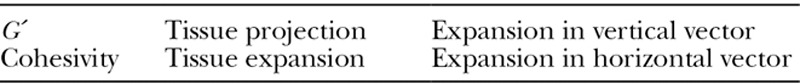
Effects from Elastic Modulus (G´) and Cohesivity

## HD FILLERS

**Fig. 1.**
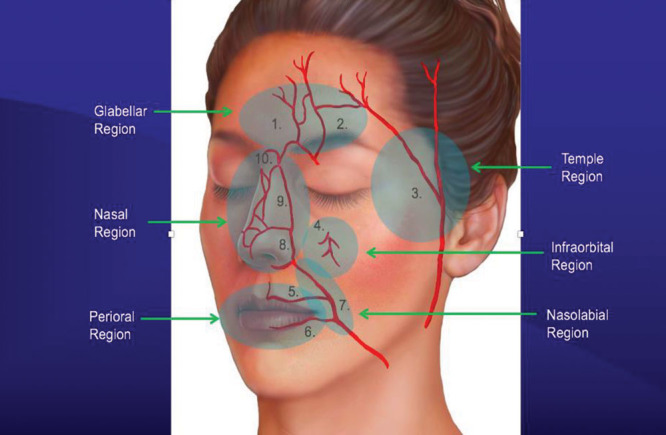
Fillers listed by ascending elastic modulus (*G*´).

HA is the most commonly used soft-tissue filler. This is due in part to the wide portfolio of products available, ease of use, low overall complication profile, and reversibility.^15^ Table 3 displays an overview of the common fillers approved in the United States. Figure 1 displays the products by *G*´. The first HA-based filler approved for use in the United States was Restylane. Originally compared with collagen-based fillers, now most formulations are compared with Restylane as a control.

**Table 3.**
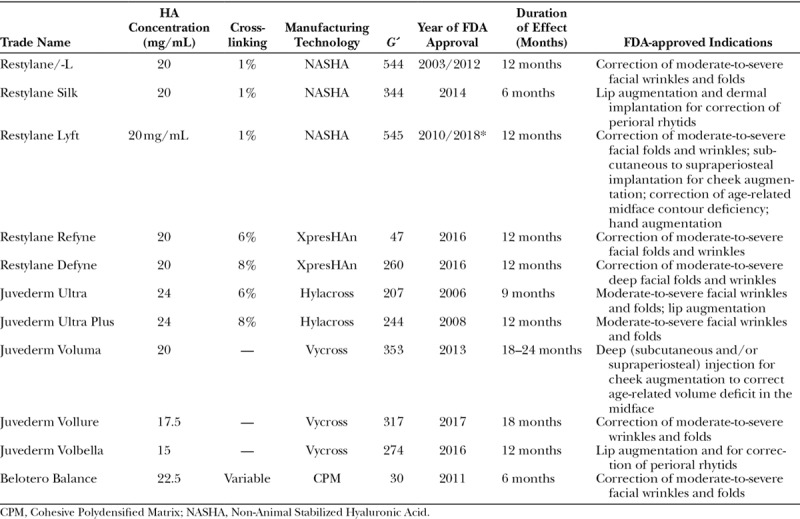
Characteristics of Hyaluronic Acid Fillers

The Restylane products are developed with the patented Non-Animal Stabilized Hyaluronic Acid technology. All first-generation Restylane gels are concentrated with 20 mg/mL of HA and have 1% of cross-linking. Each Restylane product is created by a sieving process, which allows for unique particle sizing adapted for the clinical indications of the final product.^3^ Superficially placed Restylane products contain smaller particle sizing compared with larger particles for deeper placement. The current FDA-approved first-generation Restylane products are Restylane, Restylane-L, Lyft, and Silk.

Galderma introduced 2 new products, such as Refyne and Defyne, in 2016. The second generation is formulated by a patented XpresHAn technology, which is marketed for improved dynamic function in areas of expression. Like the original Restylane line, HA concentration is 20 mg/mL; however, they differ in the cross-linking and sieving processes. They further classify these 2 products based on flexibility, or the amount of stretching a gel can withhold without breaking. Strain is used as an index of flexibility and is inversely related to cross-linking. The separate technologies (Non-Animal Stabilized Hyaluronic Acid and XpresHAn) produce dichotomous products with unique characteristics.

Juvederm (Allergan) is another line of HA fillers first gaining FDA approval in 2006. The first generation of Juvederm products was created with a patented Hylacross technology creating Ultra and Ultra Plus. This process generates variable and highly cross-linked HA from 100% of high molecular weight chains with 24 mg/mL of HA. The high HA concentration and cross-linking attribute to the high water absorption in this line.^16^

**Fig. 2.**
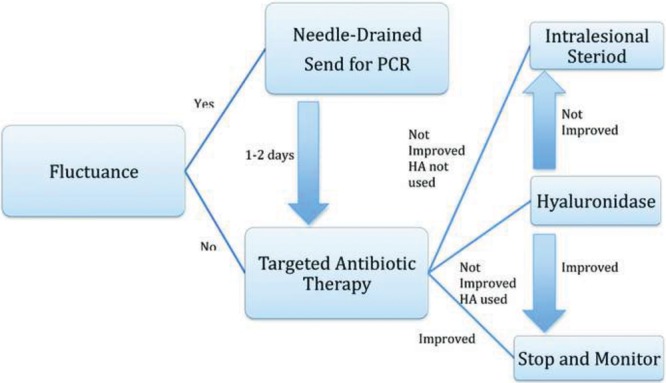
Water absorption of Juvederm line.

The second generation of fillers was created with a patented Vycross technology. This uses a proprietary blend of high and low molecular weight HA chains that are tightly cross-linked. Because the HA chains are made of a unique cross-linked blend, the products absorb less water.^17^ Fillers in this class include Voluma, Vollure, and Volbella. Maximum water absorption of Juvederm fillers is displayed in Figure 2. Overall, they have lower cohesivity than the first generation with varying HA concentrations. Voluma has the highest *G*´ of the HA fillers, whereas Vollure is the least cohesive and allows for superficial placement.

Belotero Balance (Merz Aesthetics, Greensboro, N.C.) is a HA filler approved in 2011. HA content is 22.5 mg/mL. It is formulated by patented technology called Cohesive Polydensified Matrix. This process uses a second cross-linking stage with the addition of non–cross-linked HA.^18^ Cross-linking is variable with a higher content of non–cross-linked HA. This produces a highly cohesive, yet smooth product.^19^ *G*´ is lower than other HA products making it appropriate for intradermal or superficial subdermal injection.^20^ Injection technique is advocated with serial puncture and a superficial blanching technique.^19^ Tyndall effect, bluish hue of skin from superficial placement of HA products, is thought to be avoided owing to the products low viscosity and thin consistency.

Currently, a new HA filler is under study in the Unites States, marketed as Teosyal (Teoxane, UK). It consists of monophasic HA at a concentration of 23 mg/mL. It is marketed as having reduced protein and bacterial endotoxin, resulting in less hypersensitivity reactions. Juvederm recently trademarked a new product, Volux for deep soft-tissue augmentation. It has 25 mg/mL HA concentration and is under investigation for chin and jawline augmentation.

## INJECTION BY REGION

When evaluating a patient for filler injection, the face is divided into anatomical different regions. Although each region differs slightly in terms of injection technique, the goal in every region is to avoid danger zones that could lead to skin necrosis or visual loss.^21,22^ The senior author (RJR) presents some of the preferred FDA-approved fillers by region based on the desired depth of injection, desired result, and clinical experience.

### Glabella/Forehead

The glabella is the most common site complicated by visual loss as the supratrochlear and supraorbital vessels lie superficial in this region and provide for retrograde flow to the ophthalmic artery.^23^ Neuromodulators are typically used in this region; however, deep wrinkles or the region just above the eyebrows, where neurotoxins cannot be injected, may be treated with filler.^24^ Low *G*´ fillers such as Silk, Belotero, Vobella, Refyne, and blended Juvederm (1:1 with lidocaine) are preferred by the author as superficial placement is mandatory to prevent complications. Serial puncture is advocated with crosshatching to adequately efface the line. For forehead augmentation, the author prefers an intermediate *G*´ filler such as blended Ultra Plus and Vollure. The supratrochlear and supraorbital arteries are more superficial, approximately 2 cm above the orbital rim; thus, a preperiosteal injection plane is advocated.^25^ Gentle massage can smooth the contour (**see video, Supplemental Digital Content 1**, which displays filler injection to the glabella with the serial puncture technique. It also displays forehead injection of the inferior most wrinkles, those that cannot be addressed reliably with neuromodulators. This video is available in the “Related Videos” section of the Full-Text article at PRSGlobalOpen.com or at http://links.lww.com/PRSGO/B128).

**Video Graphic 1.**
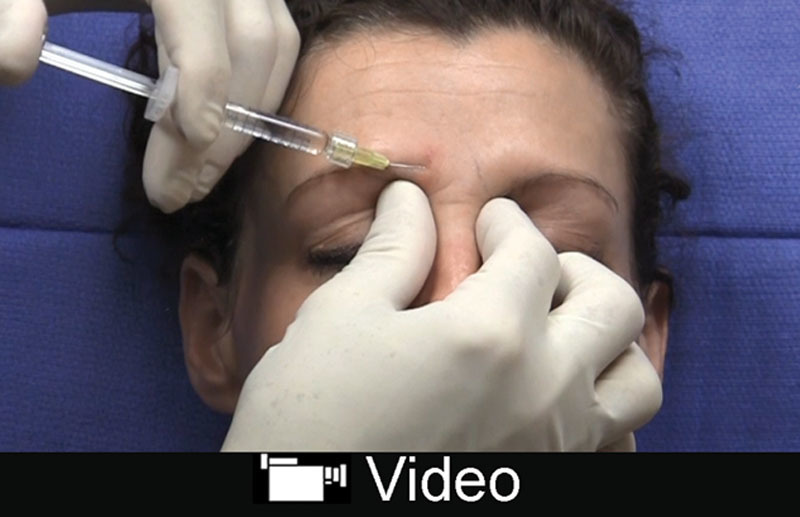
See video, Supplemental Digital Content 1, which displays filler injection to the glabella with the serial puncture technique. It also displays forehead injection of the inferior most wrinkles, those that cannot be addressed reliably with neuromodulators. This video is available in the “Related Videos” section of the Full-Text article at PRSGlobalOpen.com or at http://links.lww.com/PRSGO/B128.

### Temple

Temporal hollowing is a common age-related finding.^26^ Youthful temples are slightly convex with a smooth transition to the forehead and cheek. The frontal branch of the superficial temporal artery and the middle temporal vein must be avoided. The superficial temporal artery lies within the temporoparietal fascia starting at the root of the helix and travels superficially to above the lateral eyebrow.^27^ The middle temporal vein lies within the temporal fat pad.^28^ The author prefers a superficial subdermal injection with Vollure, blended Restylane-L, or Juvederm to avoid vasculature (**see video, Supplemental Digital Content 2**, which displays injection to the temple with blended Restylane in a superficial plane. This video is available in the “Related Videos” section of the Full-Text article at PRSGlobalOpen.com or at http://links.lww.com/PRSGO/B129). Deeper injections can be performed but must be placed 2.5 cm above the arch to avoid vascular injury.^29^

**Video Graphic 2.**
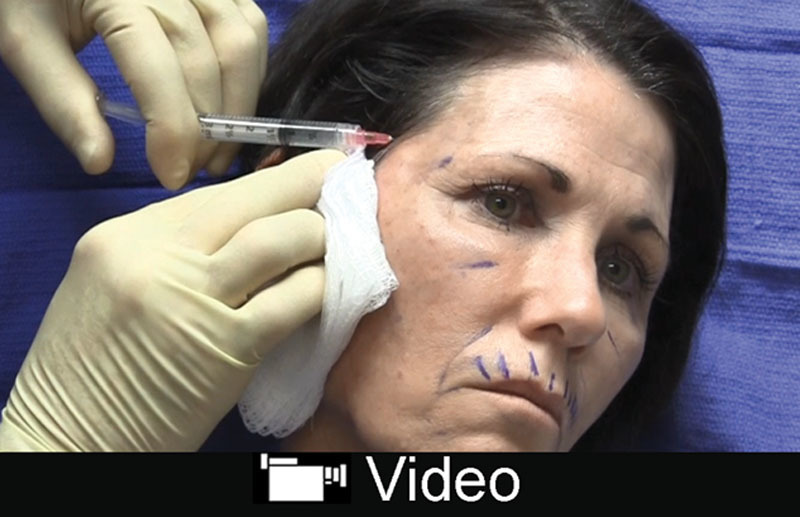
See video, Supplemental Digital Content 2, which displays injection to the temple with blended Restylane in a superficial plane. This video is available in the “Related Videos” section of the Full-Text article at PRSGlobalOpen.com or at http://links.lww.com/PRSGO/B129.

### Cheek

A youthful cheek exhibits a smooth convexity from the lower eyelid to the lower face resembling an ogee curve. Aging results in volume loss and unfavorable shadowing. Augmentation of this region should target the deep medial and lateral malar compartments. One should be mindful of the infraorbital bundle, which lies one fingerbreadth below the orbital rim at the medial limbus.^24^ The author prefers a high *G*´ product such as Voluma, Vollure, Defyne, and Lyft. Injections should be placed deep in an anterograde and retrograde manner above the periosteum at 3 major points: lateral cheek, anterior cheek, and medial cheek. Bolus and pillar injections should be avoided as this is associated with late granulomas. The product can be gently massaged (**see video, Supplemental Digital Content 3**, which displays injection to the cheek using Voluma. This video is available in the “Related Videos” section of the Full-Text article at PRSGlobalOpen.com or at http://links.lww.com/PRSGO/B130).

**Video Graphic 3.**
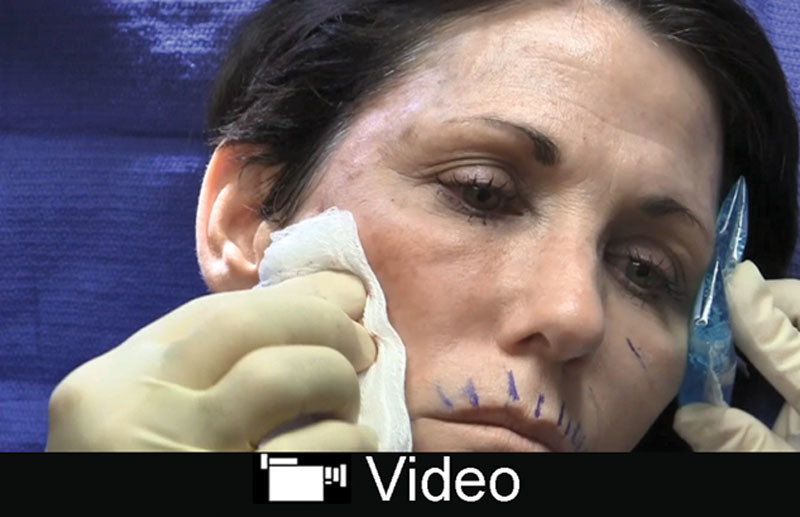
See video, Supplemental Digital Content 3, which displays injection to the cheek using Voluma. This video is available in the “Related Videos” section of the Full-Text article at PRSGlobalOpen.com or at http://links.lww.com/PRSGO/B130.

### Tear Trough

The tear trough is an unforgiving area given the thin skin and racial variability. The best candidates are those with good skin tone and minimal skin excess.^30^ The author prefers injection of a low *G*´ filler such as Restylane-L, Silk, Belotero and Vollure if placed deep.

Limit use with hydrophilic fillers as they can cause unfavorable water absorption. A linear threading technique is advocated from medial to lateral in the suborbicularis plane to minimize the chance of Tyndall effect. Attention must be paid to adequately fill the lateral hollow. Cheek augmentation aids in tear trough correction as it helps support the lower lid. Overcorrection should be avoided.

### Lips

The lips are a popular injection site for augmentation. The superior and inferior labial arteries course deep within the lip, between the orbicularis oris and the mucosa.^31^ Injections should be limited to a depth of 3 mm to avoid vasculature. Anatomically, the upper lip should measure 1/3 total vertical distance and the lower lip 2/3. The upper lip should protrude 1–2 mm anterior than the lower lip. The author prefers an algorithmic approach to lip augmentation with 5 key steps listed in Table 4.

**Table 4.**
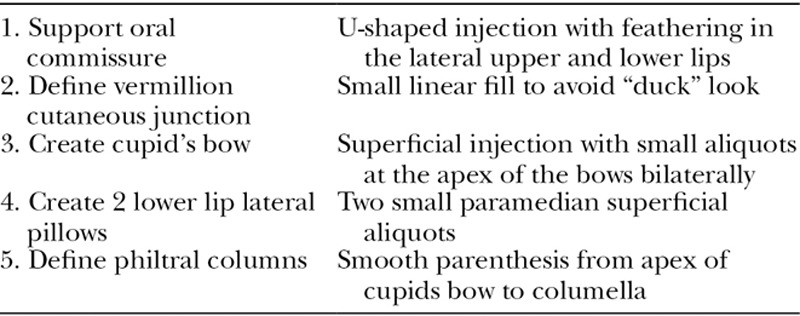
Five-step Lip Augmentation

Ultra Plus is preferred by the author; however, Ultra, Silk, Refyne, and Vollure can be used. Similarly, for perioral lines, an intermediate *G*´ filler can be used. Deeper lines tolerate the Juvederm products better because of the increased water absorption. Injection should be superficial parallel and perpendicular to the line. Overcorrection should be avoided (**see video, Supplemental Digital Content 4**, which displays injection to perioral wrinkles and lip augmentation with Restylane Silk. This video is available in the “Related Videos” section of the Full-Text article at PRSGlobalOpen.com or at http://links.lww.com/PRSGO/B131).

**Video Graphic 4.**
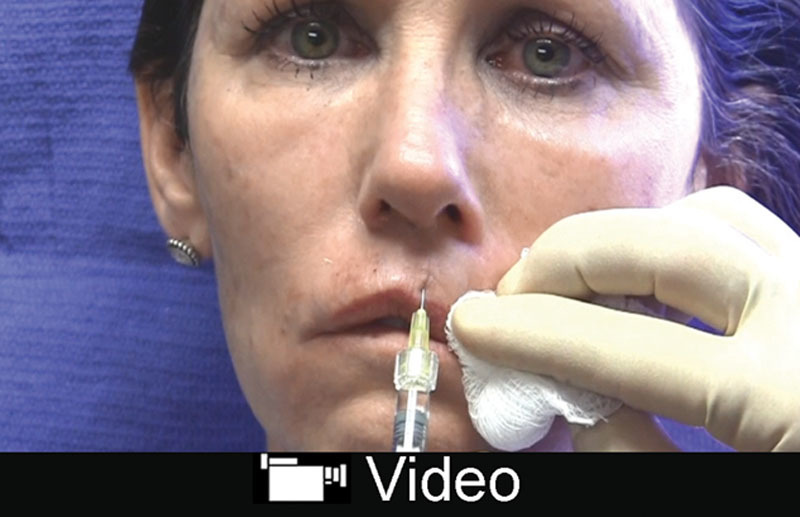
See video, Supplemental Digital Content 4, which displays injection to perioral wrinkles and lip augmentation with Restylane Silk. This video is available in the “Related Videos” section of the Full-Text article at PRSGlobalOpen.com or at http://links.lww.com/PRSGO/B131.

### Nasolabial Fold

The nasolabial fold represents a significant danger zone for augmentation related to the facial artery.^32^ The facial artery has a tortuous course; the lower two thirds of the artery travels within the muscle or deep subcutaneous tissue. It is superficial in the upper 1/3 of the nasolabial fold and ramifies with the inferior alar artery and lateral nasal artery.^32,33^ The author feels that periapical hypoplasia accounts for a significant proportion of prominent folds, especially toward the proximal 1/3 of the nasolabial fold. The author prefers a high *G*´ filler such as Vollure or others such as Restylane-L, Lyft and Voluma injected in the preperiosteal plane along the pyriform aperture (**see Video, Supplemental Digital Content 5**, which displays filler injection to the oral commissure and nasolabial fold. This video is available in the “Related Videos” section of the Full-Text article at PRSGlobalOpen.com or at http://links.lww.com/PRSGO/B132). Many different fillers are advocated for the lower 2/3 of the nasolabial fold and are dependent on the severity. It is important to inject medial to the fold in the subdermal plane as injection within the fold can make it more prominent.

**Video Graphic 5.**
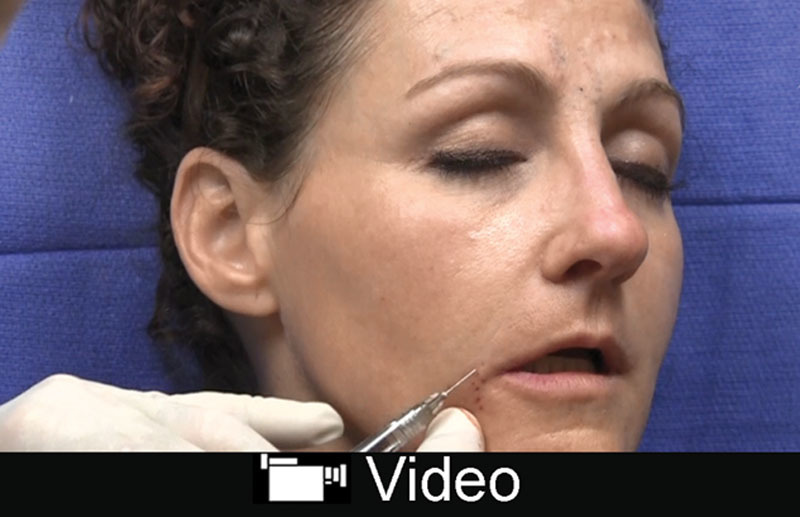
See video, Supplemental Digital Content 5, which displays filler injection to the oral commissure and nasolabial fold. This video is available in the “Related Videos” section of the Full-Text article at PRSGlobalOpen.com or at http://links.lww.com/PRSGO/B132.

### Nose

The nose is a highly vascular region of the face with the predominance of the vascular structures lying along the nasal side walls and dorsum including the lateral nasal artery, angular artery, and dorsal nasal artery.^32^ The vasculature lies above the superficial musculoaponeurotic system in the subdermal plane.^34^ One must be wary of scarred regions as the vasculature may be previously affected resulting in skin compromise.^35^ For dorsal augmentation, to camouflage irregularity or a hump, the author prefers Restylane-L placed midline in the deep preperichondrial or preperiosteal plane. The Restylane products overall absorb less water, and this is idea for the dorsum. The tip and ala are prone to necrosis secondary to compression injury or vascular injury. The author prefers Ultra or Ultra Plus for this region with a careful incremental approach using serial puncture. Filler can be layered deep within the tip to act a columellar strut. Small volumes can be used to even out alar rim irregularities or retraction. The patient is always reassessed 15 minutes after injection to ensure no vascular compromise (**see video, Supplemental Digital Content 6**, which displays injection of the nasal dorsum, middle vault and tip with Restylane. This video is available in the “Related Videos” section of the Full-Text article at PRSGlobalOpen.com or at http://links.lww.com/PRSGO/B133).

**Video Graphic 6.**
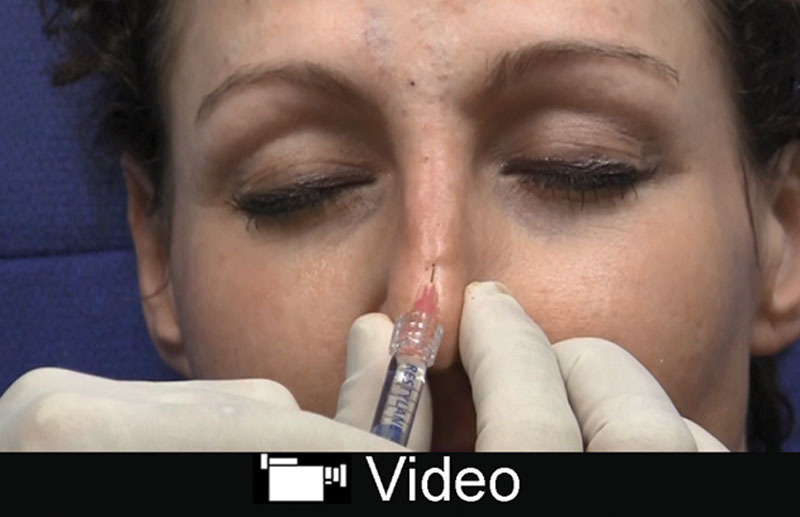
See video, Supplemental Digital Content 6, which displays injection of the nasal dorsum, middle vault and tip with Restylane. This video is available in the “Related Videos” section of the Full-Text article at PRSGlobalOpen.com or at http://links.lww.com/PRSGO/B133.

### Chin

Chin aesthetics can affect the appearance of the nose and the neck. Chin projection varies between sex. In males, the chin should be in line with the lower lip. In females, the chin should be 1–2 mm behind the lower lip.^36^ Augmentation can only address mild-to-moderate retrusion (4–10 mm), but it can have a powerful effect. The author prefers a high *G*´ filler such as Voluma, Lyft, and Defyne injected in the preperiosteal plane. Chin augmentation should not extend beyond the medial canthus. The filler should fan laterally to the prejowl sulcus for adequate blending.

## COMPLICATIONS

Fortunately, complications from soft-tissue fillers are rare given the advanced safety profile of many of the currently used products.^37^ Rohrich et al^38^ devised a classification profile to assist in management. Complications are categorized as early (less than 14 days), late (14 days to 1 year), and delayed (greater than 1 yea)r (Table 5).

**Table 5.**
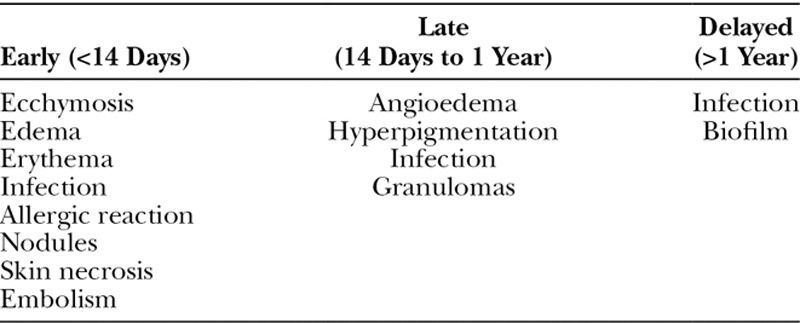
Filler Sequela/Complications

### Early

Early sequela are common and are typically technical in nature or inflammatory. Erythema, edema, and bruising are common and more frequent with more superficially placed fillers.^7^ Lumps or bumps are typically related to inadvertent superficial placement. Infections are uncommon but can be bacterial, viral, or fungal in nature.^39^ Bacterial infections such as cellulitis and abscesses are likely caused by skin flora such as *Staphylococcus* and *Streptococcus* species. Patients with a history of cold sores may be pretreated with acyclovir to prevent an outbreak.

The most feared early complications are tissue necrosis or embolism (blindness or stroke), related to intra-arterial injection.^23^ Except for blindness, vascular occlusion occurs in up to 3 out of 1,000 and is more common in non-HA fillers.^40^ Signs and symptoms can be discrete but include pain out of proportion or blanching.

**Fig. 3.**
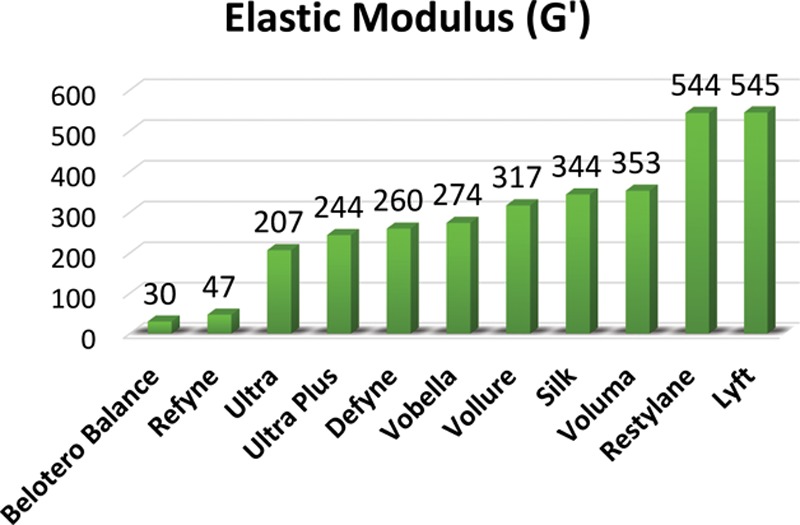
Facial danger zones. 1. Supratrochlear artery. 2. Supraorbital artery. 3. Superficial temporal artery. 4. Infraorbital artery. 5. Superior labial artery. 6. Inferior labial artery. 7. Facial artery. 8. Angular artery. 9. Lateral nasal artery. 10. Dorsal nasal artery. Adapted with permission from *Plast Reconstr Surg* 2017;139:1103.

Six facial danger zones have been described, which increase the risk of vascular compromise.^32^ These include the glabella, temple, infraorbital region, lips, nasolabial fold, and nose (Fig. 3).^41^ These areas have named arterial branches, which deserve anatomic considerations and caution when injecting in these regions to avoid serious complications. General principles for safe filler injection are listed in Table 6.

**Table 6.**
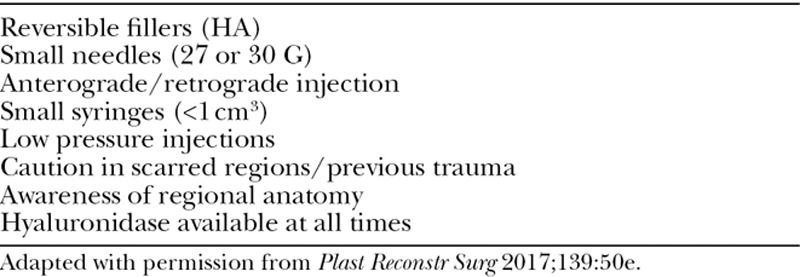
Principles for Safe Filler Injections

### Late

Late complications are usually related to foreign body granuloma reaction.^42^ A granuloma is an organized mass of inflammatory cells.^43^ This is thought to be related to the encapsulation of unfamiliar material with ongoing inflammation. The cause is theorized to result from any combination of volume of implanted material, larger filler particles, impurities, and low-grade infections.^38^ It is estimated to occur on average of 0.01–0.1%.^44^ Diagnosis is based on clinical findings of delayed swelling, erythema, or discoloration after an uneventful injection period.^45^

### Delayed

Delayed filler complications are thought to be related to biofilms. Biofilms are heterogeneous structures of bacterial colonies with an adherent extracellular matrix, which can evade an immune response.^46^ The clinical picture of a granuloma may be challenging to distinguish between inflammatory and biofilm; however, reports of delayed granulomas with preceding infectious event support an infectious diagnosis.^43^ Algorithms have been established to aid in the diagnosis and proper management of this presentation.^38^

## PREVENTION

The best management of a complication is prevention. Injectors should understand the anatomy, product, and indications for use. The FDA has set guidelines for approved use of a certain product; however, off label use is still acceptable based on clinical judgment. The author recommends aseptic technique during injections and uses chlorhexidine skin prep to decrease bioburden.

Appropriate placement can minimize palpable lumps or nodules; however, despite appropriate technique, contour irregularities may develop.^39^ Injection technique is dependent on injector preference and anatomical region. For superficial injections, a linear threading technique is advocated with small aliquots of product in an anterograde and retrograde fashion. Fanning or crosshatching may also be advised to allow even distribution of product. With deeper placement, a depot injection technique has been traditionally recommended.^47^ However, it has been the experience of the senior author that depot injections are more likely to develop delayed granulomatous reactions.^48^ Towering or thin layering can be used for deeper placement.^49^ One must always be cognizant of the needle tip location to prevent inadvertent placement. Lastly, anatomy and facial danger zones must be respected to ensure safe delivery in the targeted plane.

## TREATMENT OF COMPLICATIONS

Complications are best managed with early recognition. For erythema, edema, and bruising, cool compresses should be used. Patients should avoid exercise for 24–48 hours to prevent progression. For early lumps and bumps that are related to inappropriate filler placement, gentle massage can be instructed. For refractory lumps, Tyndall effect, or overcorrection, hyaluronidase can be used to dissolve the product.^50^

Hyaluronidase is a naturally occurring enzyme that breaks down autologous HA and is used for the degradation of HA-based filler product. Human and animal formulations are available but differ in their preparations.^51^ Hypersensitivity reactions have been reported.^52^ The senior author uses Hylenex (human recombinant hyaluronidase) (Halozyme Therapeutics, San Diego, CA) to dissolve HA products. Vitrase (Bausch + Lomb, Rochester, NY) is the other available hyaluronidase available and is made from sheep testes. The dose is titrated to effect. Clinical effects are immediate if injected directly and can be enhanced with massage.

### Necrosis

Intravascular filler injection represents the most severe complication from filler use. Injection should be stopped immediately for any suspicion of vascular compromise. The mainstay of treatment is hyaluronidase. This should be injected diffusely within the ischemic tissue.^53^ It is not necessary to inject hyaluronidase intravascularly as the product will diffuse widely, even within local vasculature. It should be injected along the course of the artery involved and titrated to effect.^23^ Additionally, warm compresses, aspirin, and hyperbaric oxygen have been advocated.^54^

### Blindness

The embolic phenomenon is the etiology of blindness and is related to retrograde flow into the central retinal artery.^55^ Majority of ocular complications result from injection near the glabella.^56^ Unfortunately, visual recovery after embolic phenomenon is very poor as irreversible retinal damage occurs within 60–90 minutes.^57^ If vision loss occurs, expert consultation with an ophthalmologist is mandatory. Retrobulbar injection of high doses of hyaluronidase is recommended to decomplex both intravascular HA and HA in surrounding tissues. Additional treatment modalities include anterior chamber paracentesis, ocular massage, mannitol, or interventional radiologic injection of hyaluronidase within ophthalmic circulation; however, none of these modalities have been shown clinically to be effective.^58^

### Nodules

**Fig. 4.**
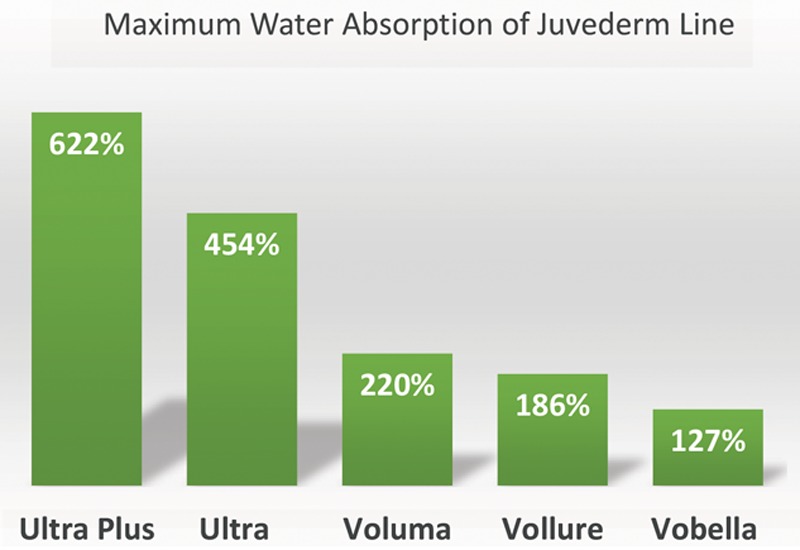
Management algorithm of late and delayed complications associated with biofilms. PCR, polymerase chain reaction. Adapted with permission from *Plast Reconstr Surg* 2014;133:865e.

Inflammatory nodules, also called granulomas, should be approached in an algorithmic manor (Fig. 4). If fluctuance is present, it should be aspirated and sent for culture. Cultures should be followed up to 21 days as atypical microorganisms may be present. A trial of antibiotics is recommended with dual drug modality such as a quinolone and a macrolide (eg, ciprofloxacin 500 mg twice daily and clarithromycin 500 mg twice daily) for 2 weeks. Hyaluronidase should never be injected with an active infection due to the risk of spreading infectious material within adjacent tissue. If no improvement, and a HA-based filler was used, hyaluronidase can be injected. If a non-HA product was used, or hyaluronidase was ineffective, intralesional triamcinolone can be given. Starting dose is 0.1 mL with 10 mg/mL concentration and titrating to effect.^59^ One must be careful with superficial placement as atrophy may result. 5-Fluorouracil is another intralesional treatment option but is considered second line.^45^ Excision should be the last in the treatment algorithm.^43^

## CONCLUSIONS

It is clear that HA-based filler products have their place in nonsurgical facial rejuvenation. Understanding basic gel properties and unique filler manufacturing process can help with the selection of an individual product and lead to more predictable results. With the plethora of products available, it is important to understand the applicability and limitations of each facial subunit injected. Safety is always of the upmost importance to minimize complications. Prevention is the key; however, if complications arise, an algorithmic approach is pivotal to success.

## ACKNOWLEDGMENT

The patient who appears in the associated videos provided written consent to be filmed.

## Supplementary Material
